# Human Influence on the Biogeochemical Reactivity of Subterranean Estuaries

**DOI:** 10.1021/acsestwater.5c01416

**Published:** 2026-05-18

**Authors:** Elisa Calvo-Martin, Xosé Antón Álvarez-Salgado, Valentí Rodellas, María José Pazó Fernández, Vanesa Vieitez Dos Santos, J. Severino P. Ibánhez

**Affiliations:** † Organic Geochemistry Lab, Department of Oceanography, Instituto de Investigacións Mariñas, Consejo Superior de Investigaciones Científicas (CSIC), Rua Eduardo Cabello 6, Vigo 36208, Spain; ‡ PhD Program in Marine Science, Technology and Management, Universidade de Vigo, Edificio de Ciencias Experimentales, Vigo 36310, Spain; § Department of Physics, Universitat Autònoma de Barcelona, Edifici C. Bellaterra, Cerdanyola del Vallès 08193, Spain

**Keywords:** submarine groundwater discharge, subterranean estuary, seepage face, anthropogenic impact, dissolved inorganic nutrients, dissolved organic matter

## Abstract

Subterranean estuaries play a key role in the land-ocean interface by modulating groundwater-borne and recycled solutes discharged to the coast. Despite their importance for coastal ecosystems, the sensitivity of these systems to human activities is still unknown. To address this gap, dissolved organic matter (DOM) and dissolved inorganic nutrients of a pristine site were compared with those from two nearby, semiurban sites characterized by contrasting oxygen conditions. The local aquifers surrounding the semiurban subterranean estuaries contained more nitrogen, silicate, and DOM of higher molecular weight than the aquifers surrounding the pristine site. Despite the different chemical composition of the arriving fresh groundwater, N/P ratios and the quantity of humic-like DOM compounds at the pristine site were intermediate between those of the two semiurban sites. This pattern reflected the intermediate permeability and oxygenation of the pristine beach, highlighting the role of the sediment matrix in modulating the exported solutes. Enhanced oxygenation at one semiurban site resulted from a human-derived gravel layer that increased sediment permeability and reduced internal residence times. The anthropogenic alteration of the sediment permeability had a greater influence on the nutrients and DOM found in subterranean estuaries than did the chemical composition of the inland aquifers.

## Graphical abstract

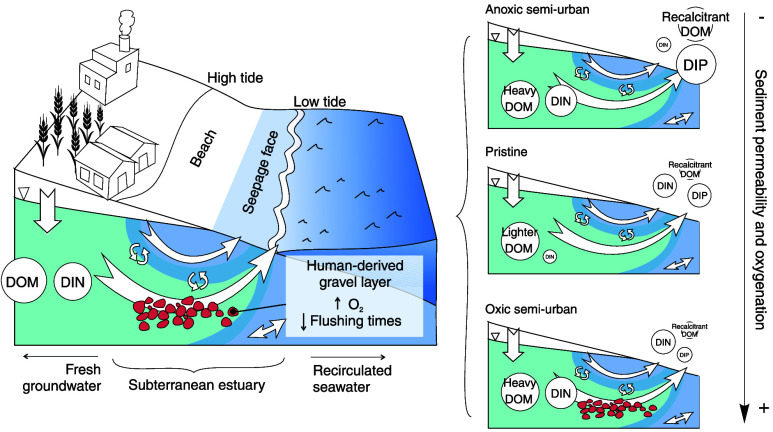

## 1 Introduction

Human activities near the coast have significantly altered natural biogeochemical cycles, leading to widespread groundwater enrichment of nutrients and dissolved organic carbon (DOC) via agriculture, animal waste, sewage, and urban leachates.^1,2^ These polluted groundwaters can reach coastal ecosystems through fresh submarine groundwater discharge (fresh SGD^3^), causing toxic algal blooms, eutrophication, and shifts in seawater nutrient limitations.^4,5^ Fresh SGD is often overlooked as a significant solute source to the coast due to its low volumetric contribution, but it can locally rival riverine solute fluxes because of its high solute concentrations.^6^ When including seawater recirculated through permeable sands (i.e., saline SGD), the primary component of SGD, SGD can rival or even exceed river volumetric and solute fluxes globally.^7^

Beaches typically represent the seaward boundary of coastal aquifers, where fresh groundwater mixes with intruding seawater.^8^ These zones are known as subterranean estuaries.^9,10^ Tides and waves pump dissolved oxygen and coastal organic matter into the sediment, fueling intense aerobic respiration there.^11^ Seawater also introduces large amounts of sulfate into subterranean estuaries, which can act as a strong oxidizing agent under anaerobic conditions.^12^ Coastal particulate organic matter (POM) is processed at shallow sediments, and coastal dissolved organic matter (DOM) supports the deeper heterotrophic community, releasing dissolved inorganic nitrogen (DIN), dissolved inorganic phosphate (DIP), and other reduced byproducts to porewaters.^11,13,14^ Thus, infiltrating seawater provides new electron acceptors, inorganic nutrients, and fresh organic substrates to the subterranean estuary, while fresh groundwater typically transports anthropogenic nutrients, terrestrial aromatic DOM, and silicate.^15–17^ With this underground admixture, intense biogeochemical transformations occur in subterranean estuaries, significantly modulating groundwater solute content prior to discharge to the coast.^18^

Subterranean estuaries can naturally mitigate anthropogenic DIN enrichment of coastal groundwaters before they reach coastal waters via denitrification and anaerobic ammonium oxidation (Anammox) but also enhance DIN fluxes via organic matter processing, dissimilatory nitrate reduction to ammonium, and/or nitrification.^19,20^ Anthropogenic nitrogen inputs via continental groundwaters also accelerate organic carbon turnover, enhancing humic-like DOM production.^21,22^ Anthropogenic DOM inputs feed the heterotrophic metabolism of the subterranean estuary, driving DOM transformations.^23^ Groundwater overpumping reduces freshwater supply and promotes seawater intrusion, increasing pH.^24^ This releases DIP and NH_4_^+^ adsorbed onto the sediment, fueling nitrification and microbial assimilation there.^19,25^ Land reclamation also alters the magnitude and geometry of infiltrating seawater, consequently modifying porewater residence times.^26^ The influx of human-impacted fresh groundwater, along with the chemical and physical alteration of their circulation zones, may reshape the internal reactivity of the subterranean estuaries, affecting the quantity of solutes discharged in ways that remain poorly understood.

This study aims to assess the human influence on the biogeochemistry of subterranean estuaries. To do so, the composition and reactivity of two semiurban subterranean estuaries of contrasting redox conditions were compared with a subterranean estuary located in a pristine area, which, though under similar hydrological and oceanographic regimes, is subject to minimal anthropogenic influence.

## 2 Materials and Methods

### 2.1 Site Description

This study was performed in three mesotidal beaches at the Ría de Vigo (NW Iberian Peninsula): the pristine Figueiras Beach, at the northern Cíes Islands, and the semiurban Panxón and Ladeira beaches, located in Baiona Bay (Figure 1A,B). Despite their proximity (∼3 km), Ladeira is predominantly anoxic, whereas Panxón is oxygenated due to enhanced transport through a human-originated gravel layer.^27^ Figueiras Beach is located ∼15 km from both sites.

**Figure 1:**
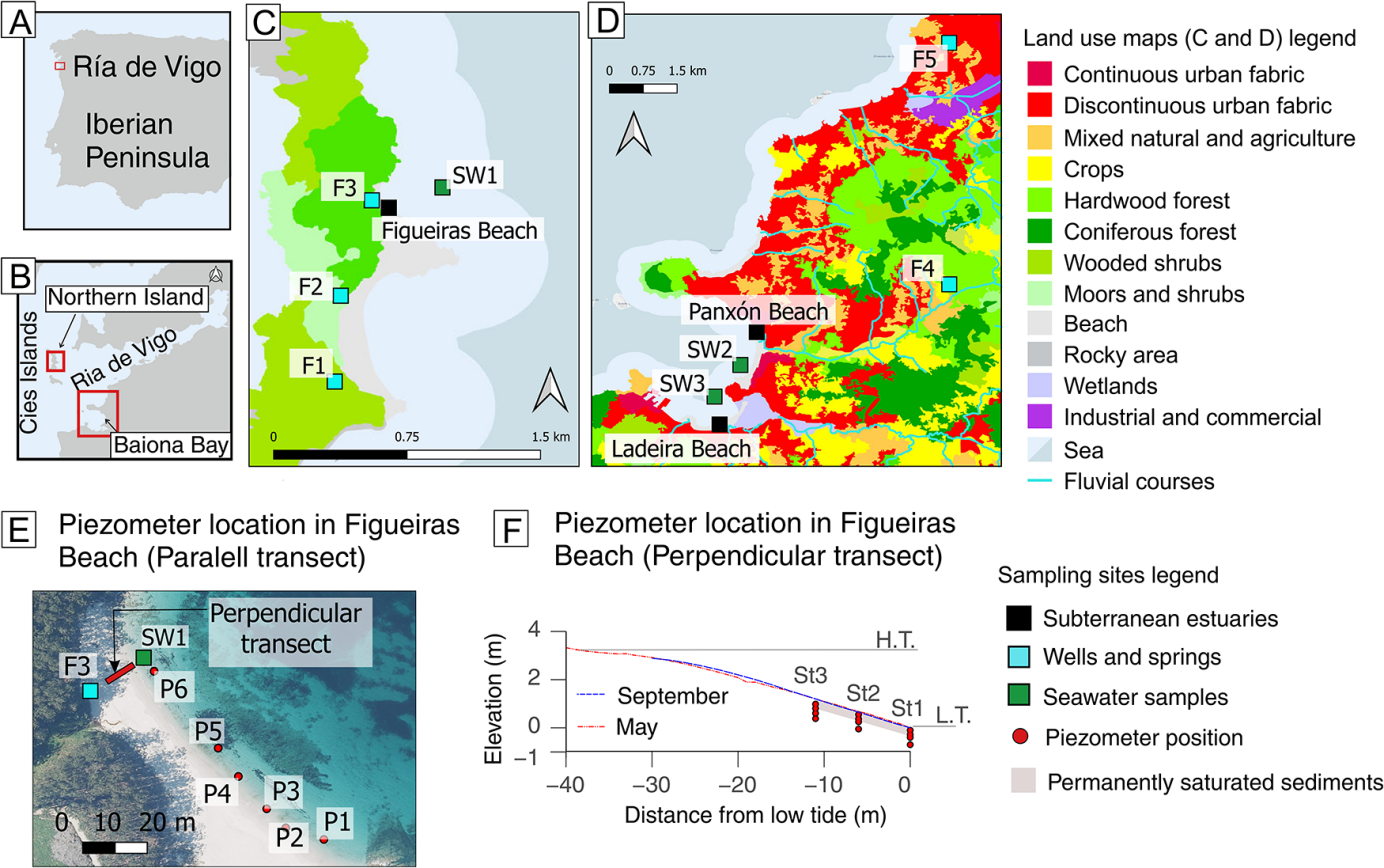
Location of the springs and wells (light blue squares), seawater (dark green squares), and subterranean estuaries (black squares) used in this study, together with the land use coverage at the Northern Cíes Islands (C) and Baiona Bay (D). The piezometers installed in Figueiras Beach (red dots) along the parallel transect are represented in part (E) and the perpendicular transect in part (F). In the perpendicular transect, the piezometer position is represented together with the beach profile measured in the September and May surveys. The satellite map was obtained from Sentinel 2 (dataspace.copernicus.eu). Land use coverage was obtained from the Corine Land Cover data from the Spanish National Geographic Institute (visualizadorescdn.ign.es/SIOSE).

The Ría de Vigo is a V-shaped coastal inlet affected by intermittent coastal upwelling, protected at its mouth by the Cíes Islands;^28^ Figure 1A. The climate of the Ria de Vigo is Atlantic with Mediterranean influence and characterized by mild temperatures with high precipitation from fall to spring (see Supporting Information Figure S1 for the meteorological data during the surveys). Recently, fresh SGD was identified as an important freshwater source to this coastal embayment.^29^

The geology underlying the study zone consists of highly fractured crystalline rocks, such as granites, shales, and schists.^30^ Aquifers are structured in two layers: a shallow layer within the regolith, with higher groundwater storage capacity, and a deeper layer within the fractured basement rocks, whose storage depends on the degree of fracturing.^31^ As a result, groundwater recharge is highly heterogeneous, between 1 and 44% of precipitation.^31^ Residence times within crystalline aquifers of the NW Iberian Peninsula are generally short, with an average time of semidepletion of groundwater discharge of about 23 days.^31^

Transit times between the terminus of the coastal aquifer (i.e., groundwater prior to entering the beach) in Baiona Bay and the sampled sediments of Panxón and Ladeira beaches estimated using radioisotopes ranged from 9 to 40 days;^27^ Table S1 in Supporting Information. The northern Cíes Islands has a small watershed (2.8 km^2^ of surface area^32^) compared to the Miñor River, the main fluvial course discharging into Baiona Bay, which has a watershed of ∼76 km^2^ and a mean (standard deviation) annual flow of 1.9 (1.5) m^3^/s for 2018–2019 (meteogalicia.com^33^).

The northern Cíes Islands belongs to the Maritime-Terrestrial National Park of the Atlantic Islands of Galicia. Its vegetation is dominated by shrubs, pines, and eucalyptus;^34,35^ Figure 1C. On the other hand, the lands surrounding Baiona Bay include urban, agricultural, and forest areas;^36^ Figure 1D. Although tourism is a well-developed activity in both areas, visitors to the Cíes Islands are limited during the summer and absent during fall and winter, which implies a much lower impact of this activity in Figueiras Beach compared to Baiona Bay beaches.^37^ Nevertheless, the impact of these activities cannot be evaluated with the survey design followed here.

### 2.2 Cíes Islands Surveys (Pristine Site)

Surveys took place during summer 2021 and spring 2022 in the northern Cíes Islands, focusing on Figueiras Beach (Figure 1B,E). The survey strategy did not capture the entire seasonality of the system. Nevertheless, previous studies showed consistent differences between Panxón and Ladeira beaches in nitrogen and organic carbon processing along the four seasons,^27,38,39^ suggesting that the observed differences in the biogeochemistry of the three subterranean estuaries in spring and summer might be consistent throughout the year.

Figueiras Beach porewaters were sampled using push–pull piezometers (M.H.E. Products, United States; 4 cm of screen length) connected by tubing with a field peristaltic pump (Vampire Sample Collector, Buerkle, Germany) and airtight syringes during low spring tides along two transects: one parallel and another perpendicular to the coastline (Figure 1D,E). In the parallel transect, six piezometers separated by 25 m from each other were installed within the middle of the intertidal permanently saturated zone during low tide (i.e., during active seepage) at variable depths between 35 and 50 cm (P1 to P6; Figure 1D). In the perpendicular transect, piezometers were buried in the upper, middle, and lower limits of the permanently saturated sediments at 10, 28, 44, and 70 cm (90 cm in spring 2022) depth (Figure 1E). Terrestrial groundwater was sampled at two springs (F1 and F2; Figure 1C), collected directly from the closest tap and at one spring close to the perpendicular transect location (F3; Figure 1C,D). Seawater was sampled near the perpendicular transect location during low tides using airtight syringes (Figure 1E,D).

Collected water samples were used to determine salinity, dissolved oxygen, pH, inorganic nutrients (DIN), dissolved silicate (DSi; SiO_4_H_4_ and DIP), DOC, colored (CDOM), fluorescent DOM (FDOM), and radon activities. Figueiras Beach profile was measured using an optical level (LEICA automatic level). Surface sediment samples were collected using a manual mini-corer to measure granulometry, porosity, carbonate content, total organic carbon content, and organic carbon to nitrogen ratio. Further details are described in Text S1.

### 2.3 Baiona Bay Surveys (Semiurban Sites)

Seasonal surveys were conducted between 2018 and 2019 in Baiona Bay, focusing on Panxón and Ladeira beaches (Figure 1B). In this study, only the spring and summer surveys conducted in Baiona Bay were used for comparison with Cíes Islands surveys. The similar temperatures between the sampled seasons (Supporting Information Figure S1) and the similar seasonal pattern observed in the measured porewater salinities (see 3.3) facilitate the comparison between 2018 and 2019 and 2021–2022 surveys.

Piezometers were installed in the upper, middle, and lower limits of the permanently saturated sediments (i.e., perpendicular transect) at 10, 29, 56, 86, 116, and 176 cm depth of Panxón and Ladeira subterranean estuaries following a similar layout as in Figueiras Beach (Figure 1F; Supporting Information Figure S2). No parallel transect was performed there. Seawater samples were collected close to the sampled beaches, and groundwater samples at nearby wells (screened within the regolith). Sample collection followed the same approach as in the Cíes Islands survey. For further details, see Figure S2 at Supporting Information. Additional surface sediment corers (<20 cm) were collected in summer 2023 to measure sediment size distribution of Panxón and Ladeira beaches.

### 2.4 Analytical Methods

Analytical methods for both Cies Islands and Baiona Bay surveys are described in Supporting Information, Text S2.

### 2.5 Calculations

Porewater samples were classified into the circulation zones defined by Robinson et al.^40^ using porewater salinities (Figure S3 in Supporting Information and Calvo-Martin et al.^27^). The upper saline plume refers to the zone shaped by tidally driven seawater recirculation. The freshwater discharge tube is formed below the latter by the terrestrial groundwater.^40^ The salt wedge, formed by the intrusion of seawater at the low tide, was not sampled during the Figueiras Beach surveys (Figure S3 in Supporting Information). To facilitate comparison between sites, samples from the salt wedge of Panxón and Ladeira beaches were excluded from this study.

The chemical reactivity within Panxón, Ladeira, and Figueiras beaches was evaluated by applying the estuarine conservative mixing model using salinities and ^222^Rn activities as conservative tracers^18^. Seawater samples collected nearby to the beaches were used as marine (saline) endmembers. To evaluate the reactivity of the freshwater discharge tubes, F3 spring for Figueiras Beach and F4 well for the semiurban beaches were used as terrestrial (fresh) endmembers (Figure 1B,C). These wells were chosen because of their proximity to the sampled beaches, although similar results were obtained when using other sampled springs and wells as terrestrial endmembers (Table S2 in Supporting Information). The reactivity of the upper saline plumes was evaluated using “terrestrial” endmembers, the samples with the highest radon or the lowest salinity found there at each survey. Deviation from conservative mixing (i.e., measured—conservative mixing concentrations) was used as a proxy of addition/removal processes. A detailed explanation is included in Text S3 at Supporting Information.

The absorption coefficient at 320 nm (a_320_) was used as a proxy to CDOM concentration. The SUVA_320_ index (DOC/a_320_) was calculated as a proxy of the abundance of conjugated C-double bonds,^49^ the slope between 275 and 295 nm (–S_275/295_) as a DOM molecular weight index,^50^ the C-normalized peak T (peak T/DOC) as a DOC lability index, and the C-normalized peak C (peak C/DOC) as a DOC humification index. Differences between groups were tested using the Kruskal–Wallis test. Relationships between variables were evaluated through Spearman correlations. Results are expressed in mean (standard deviation). Transit times from the terminus of the coastal aquifer and the sampling site in Figueiras Beach were calculated as in Panxón and Ladeira beaches (see Text S4 in Supporting Information^27^). Porewater residence times within shallow sediments (<30 cm depth) were calculated following Goodridge and Melack.^41^ Porewater discharge rates were estimated by dividing the sample depth by the estimated residence time, assuming vertical transport (see Text S5 in Supporting Information). All of the calculations were performed in R (v. 4.2.1).

## 3 Results

### 3.1 Chemical Composition of Continental Groundwaters and Seawater

Continental groundwaters surrounding Baiona Bay contained more NO_3_^–^ and DSi, and their DOM had a higher molecular weight than groundwaters from the northern Cíes Islands (Tables 1 and S3 in Supporting Information). Baiona Bay seawater was similar to Cíes, except for the DOC lability (peak T/DOC), which was twice as high in Cíes compared to Baiona for each season (Table S3 in Supporting Information). All of the sampled sea and continental groundwaters were well oxygenated (O_2_ > 100 μM).

**Table 1:** Median of Dissolved Oxygen, DIN, DIP, DSi, DOC, and C-Normalized Peak C (Peak C/DOC) with Their Range of Values in Brackets

	Oxygen (μM)	DIN (μM)	DIP (μM)	DSi (μM)	DOC (μM)	Peak C/DOC (×10^4^ r.u.)
Seawater						
Baiona Bay *(n* = 4)	257 (247–265)	4.6 (1.6–6)	0.4 (0.1–0.6)	8.1 (5.7–11)	88 (81–99)	28 (23–29)
Northern Cíes Islands (*n* = 2)	230 (186–274)	3.0 (1.3–4.7)	0.3 (0.2–0.3)	2.0 (1.3–2.7)	95 (86–104)	26 (25–28)
Continental Groundwaters						
Baiona Bay *(n* = 4)	216 (192–240)	280 (102–397)	4.6 (0.2–10)	71 (9.9–147)	128 (28–170)	171 (136–203)
Northern Cíes Islands (*n* = 6)	187 (111–283)	14 (0.8–20)	0.2 (0.1–2.5)	2.6 (1.5–6)	169 (124–374)	179 (6.9–235)
Upper Saline Plume						
Figueiras (*n* = 14)	58 (12–210)	33 (8.6–83)	2.4 (0.9–3)	13 (5.7–26)	84 (63–104)	84 (47–140)
Ladeira (*n* = 13)	7.8 (0–42)	25 (2.2–56)	11 (1–19)	35 (15–54)	84 (60–148)	127 (82–231)
Panxón (*n* = 14)	29 (4.2–216)	34 (3.4–100)	2.2 (0.7–4.3)	31 (3.7–49)	53 (40–80)	90 (61–140)
Freshwater Discharge Tube						
Figueiras (*n* = 21)	23 (0–89)	23 (6.2–107)	3.7 (1–6.6)	23 (12–56)	130 (92–753)	137 (40–246)
Ladeira (*n* = 15)	6 (0–42)	14 (5.2–78)	14 (1.8–40)	43 (17–117)	75 (59–124)	225 (98–450)
Panxón (*n* = 18)	158 (9.8–223)	53 (2.8–111)	2.5 (1.8–9.6)	40 (16–52)	39 (30–75)	115 (77–149)

### 3.2 Beach Profile and Sediment Characterization

The beach profile and sediment characterization of Panxón and Ladeira beaches are described in Calvo-Martin et al.^27^ Briefly, the mean (standard deviation) of the beach slopes across surveys in Ladeira was 4.1 (0.1)%, whereas Panxón beach slope was 2.8 (0.6)%. The porosity of their sediment was 0.47 (0.02) in Panxón Beach and 0.46 (0.06) in Ladeira Beach. The sediment organic matter content per dry weight of the surface sediment of Panxón and Ladeira Beach was 1.33 (0.10)% and 0.93 (0.40)%, respectively. Their sediment organic C/total N ratio was 3.2 (0.8) for Panxón and 10.7 (3.5) for Ladeira.

The beach profile of Figueiras Beach was composed of a surf zone with a gentle slope (∼10%). Its uppermost station (St3) presented lower porosity (0.39 (0.03)), lower organic matter content (0.84 (0.16) % organic matter in dry weight) and a higher organic C/total N ratio (4.7 (0.7) mol C/mol N) than the lowermost station (St1; a porosity of 0.46 (0.03); an organic matter content of 1.39 (0.17) % organic matter in dry weight; an organic C/total N ratio of 3.92 (0.36) mol C/mol N). Figueiras presented low carbonate content (2.72 (0.97) %). The mean (standard deviation) grain sizes of the surface sediments of Figueiras, Panxón, and Ladeira were 0.38 (0.16), 0.19 (0.04), and 0.16 (0.03) mm, respectively.

### 3.3 Description of the Subterranean Estuary of Figueiras Beach

Radon-enriched (3.3 to 82 kBq m^–3^), brackish porewaters in deep sediments (>50 cm) indicate mixing of seawater and fresh groundwater, forming a freshwater discharge tube within Figueiras Beach. A significant negative correlation between ^222^Rn and salinity (Spearman’s correlation (ρ): summer: −0.81; spring: −0.75) suggests that brackish waters originate from continental groundwaters and that there is a minimal influence of ^222^Rn production and decay within the subterranean estuary. Porewaters with higher salinities were situated over the radon-enriched porewaters, shaping an upper saline plume (salinity >33.5; Figure S4 in Supporting Information). Transit times between the terminus of the coastal aquifer and the sampled sediments of Figueiras Beach were 1.1–39.0 days, within the range found at Panxón and Ladeira beaches (Table S1). Monthly accumulated rainfall prior to the summer survey (65.9 L m^–2^ day^–1^) was similar to that of the spring survey (70.6 L m^–2^ day^–1^), while air temperatures were higher in summer (18.9 °C) than in spring (16.2 °C; Figure S1). However, salinities in the Figueiras freshwater discharge tube were lower during summer (minimum: 26.7) than in spring (minimum: 31.4; Table S4 and Figure S4 in Supporting Information).

Figueiras Beach freshwater discharge tube was predominantly suboxic (<60 μM) and more saline (median salinity of 32.0; Table S3), compared to the fresher and oxygenated Panxón and anoxic Ladeira freshwater discharge tubes (Table 1; median salinities of 27.7 and 26.7, respectively, Table S3 in Supporting Information). As in Figueiras Beach, the minimum salinities reached at the freshwater discharge tube in Panxón and Ladeira beaches were similar or lower in summer than in spring (Table S4 in Supporting Information). Dissolved oxygen levels in the upper saline plume of Figueiras were comparable to those at Panxón and significantly higher than those at Ladeira (Kruskal–Wallis test *p* < 0.05; Table 1). No significant differences were observed between surveys (*p* > 0.05), except for the higher oxygenation in Ladeira upper saline plume during spring compared to summer (*p* < 0.05).

### 3.4 Inorganic Nutrients Content and Reactivity

Groundwater-borne nitrogen, mainly as NO_3_^–^ (Table S5; ^222^Rn and DIN Spearman’s ρ: 0.53), was detected within the freshwater discharge tube of Panxón beach (Table 1). Part of this DIN was attributed to apparent production along the flow path (Figure 2). Yet, DIN concentrations in the freshwater discharge tube of Figueiras were comparable to those at Panxón (Kruskal–Wallis test *p* > 0.05), although the median concentrations were still higher in the oxygenated semiurban beach (Table 1). NO_3_^–^ and NH_4_^+^ levels in the freshwater discharge tube of Figueiras were above conservative mixing (Figure 2). Groundwater-borne nitrogen was absent in the freshwater discharge tube of Ladeira due to apparent removal in transit, although deviation from conservative mixing indicated production of NH_4_^+^ (Figure 2).

**Figure 2:**
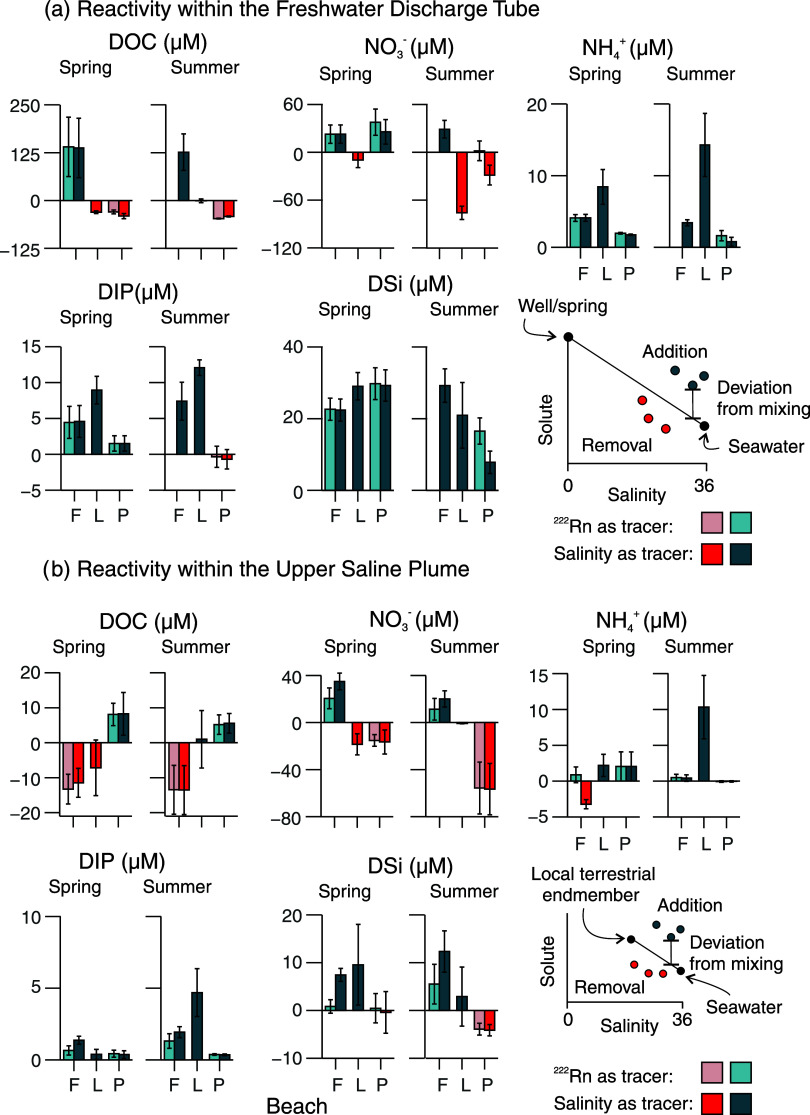
Mean deviation from conservative mixing at Figueiras (F), Ladeira (L), and Panxón (P) beaches calculated with radon (light colors) and salinity (dark colors) for (a) the freshwater discharge tube and (b) the upper saline plume. The error bar represents the standard error.

DIN content in the upper saline plume was similar across beaches (Kruskal–Wallis test *p* > 0.05; Table 1). These similarities hide a contrasting nitrogen processing (Figure 2). NO_3_^–^ removal dominated Panxón shallow sediments; whereas in Ladeira, DIN was explained by a balance between in situ NH_4_^+^ production and NO_3_^–^ removal. DIN in the upper saline plume of Figueiras was likely produced in situ through NO_3_^–^ production (Figure 2). The apparent NO_3_^–^ removal within the upper saline plume of Panxón and the freshwater discharge tube of Ladeira was higher in summer than in spring (Kruskal–Wallis *p* < 0.05; Figure 2). The apparent production of NH_4_^+^ in Ladeira peaked in summer for both circulation zones (*p* < 0.05; Figure 2). NH_4_^+^ was negatively correlated to O_2_ in Ladeira (ρ:-0.58) and Figueiras (ρ:-0.73) and to NO_3_^–^ in Figueiras (ρ:-0.77) and Panxón (ρ:-0.53).

The three beaches accumulated DIP in both circulation zones, as suggested by porewater DIP concentrations above conservative mixing (Figure 2). Porewater DIP was linked to organic matter processing, as shown by significant correlations of DIP with peak C/DOC and DOC in the three beaches (Spearman’s *p* < 0.05). Apparent DIP production was higher in Ladeira, concomitant with its higher porewater DIP concentrations (Table 1, and Figure 2). Summer also showed the higher apparent DIP production compared to spring in Ladeira (*p* < 0.05; Figure 2).

DSi in the three beaches was associated with the terrestrial endmember (Spearman’s correlations of ^222^Rn vs DSi in Figueiras: 0.63; Ladeira: 0.74; salinity vs DSi in Panxón: −0.58; *p* < 0.05). The higher median DSi concentrations of the freshwater discharge tubes of Panxón and Ladeira compared to Figueiras are in accordance with the higher DSi concentrations of their terrestrial endmembers (Table 1). DSi seemed to accumulate in the freshwater discharge tube of the three beaches and in the upper saline plume of Figueiras Beach (Figure 2). The upper saline plumes of Panxón and Ladeira did not show any apparent addition/removal pattern of DSi (Figure 2).

As a result of their contrasting reactivity, the three subterranean estuaries showed distinct nutrient stoichiometry (Figure 3). Using as a reference the Redfield N/P/Si molar ratio of 16:1:15,^51^ most porewater samples fell into the DIP-enriched category (62% of the samples), 31% into the DIN enrichment and only 6% into the DSi-enrichment (Figure 3). DIN enrichment only dominated in the freshwater discharge tube of Panxón (Figure 3). The percentage of DIN-enriched samples was higher in Figueiras (26%) than in Ladeira (10%; Figure 3). The DSi/DIP ratio showed little standard deviation within the same beach compared to those ratios related to DIN (Figure 3). Panxón showed the largest DSi/DIP ratio (median: 14.3), followed by Figueiras (6.8) and Ladeira (3.7; Figure 3). Nutrient ratios were similar among the surveys, except the DIP/DSi ratio in Ladeira, which was higher in spring (5.9) than in summer (3.0; Kruskal–Wallis *p* < 0.05).

**Figure 3:**
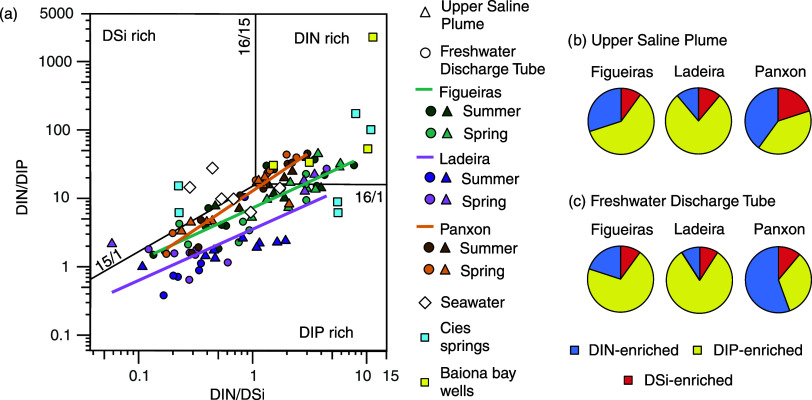
(a) DIN/DIP versus DIN/DSi from porewater, seawater, and continental groundwater samples. Dot symbols within porewater samples represent the circulation zone, and the colors represent the beach and season. (b, c) Fraction of the samples from the upper saline plume (b) and freshwater discharge tube (c) that are DIN-enriched, DIP-enriched, or DSi-enriched compared to the Redfield ratio. Regression lines were plotted for the beach samples (Spearman’s *p* < 0.05).

### 3.5 DOM Content, Composition, and Reactivity

Although the sampled continental endmembers showed similar DOM content, the freshwater discharge tube of Figueiras contained two to eight times more DOC than both semiurban sites (Table 1). DOC at Figueiras was mainly supplied by continental groundwater (Spearman’s correlation salinity vs DOC ρ: −0.79; *p* < 0.05), whereas in the semiurban beaches, it was associated with recirculated seawater (*p* < 0.05; Panxón ρ: 0.68; Ladeira ρ: 0.51). Yet, deviation from conservative mixing suggests an active production of DOC and peak C along the terrestrial groundwater flow path in Figueiras and an active DOC consumption at both semiurban sites (Figures 2 and S5 in Supporting Information). Porewater peak T levels were above conservative mixing concentrations at the three beaches (Figure S5 in Supporting Information). DOM recalcitrance peaked in the freshwater discharge tube of Ladeira, followed by Figueiras and Panxón (Kruskal–Wallis *p* < 0.05; Table 1). The maximum levels of DOC and peak T were detected in the lower limit of the permanently saturated sediments of Figueiras Beach (Figure S6 in Supporting Information).

The upper saline plume of Figueiras contained similar DOC levels to Ladeira, and both were higher than those of Panxón beach (Kruskal–Wallis *p* < 0.05; Table 1). DOM in Ladeira upper saline plume was also more recalcitrant than that of Panxón and Figueiras (Kruskal–Wallis *p* < 0.05; Table 1). Deviation from conservative mixing suggests that there was production of DOC in the upper saline plume of Panxón Beach, whereas Figueiras subterranean estuary seemed to consume peak T and DOC (Figures 2 and S5 in Supporting Information). On the contrary, DOC in Ladeira Beach upper saline plume behaves conservatively (Figure 2).

### 3.6 Fluxes of Nutrients and DOM toward the Coast

Discharge rates during active seepage were higher in Panxón (0.37 ± 0.19 cm hour^–1^) and Ladeira (0.31 ± 0.22 cm hour^–1^) than in Figueiras Beach (0.15 ± 0.06 cm hour^–1^; Table 2). Discharge rates in Figueiras Beach showed little seasonal variability, while in Panxón and Ladeira, they peaked in spring, consistent with the higher accumulated rainfall (Figure S1).

**Table 2:** Mean of Porewater Discharge Rates and DIN, DIP, DSi, and DOC Fluxes Estimated for Each Season and Beach, Including the Number of Samples (*n*) Used to Calculate Each Mean^a^

		Discharge rate (cm hour^–1^)	DIN (μmol m^–2^ hour^–1^)	DIP (μmol m^–2^ hour^–1^)	DSi (μmol m^–2^ hour^–1^)	DOC (μmol m^–2^ hour^–1^)
Figueiras	Spring (*n* = 3)	0.17 ± 0.12	59.1 ± 41.6	2.5 ± 2.0	14.2 ± 10.3	115.7 ± 81.6
	Summer (*n* = 3)	0.13 ± 0.05	21.5 ± 13.6	2.5 ± 1.2	23.0 ± 9.3	134.3 ± 52.4
Panxón	Spring (*n* = 5)	0.45 ± 0.34	63.9 ± 56.2	10.1 ± 8.2	121.5 ± 98.2	305.9 ± 234.6
	Summer (*n* = 5)	0.29 ± 0.18	67.5 ± 49.4	12.6 ± 8.7	99.1 ± 63.9	119.7 ± 74.4
Ladeira	Spring (*n* = 5)	0.42 ± 0.31	200.9 ± 166.1	37.0 ± 29.5	94.8 ± 72.3	336.1 ± 249.3
	Summer (*n* = 2)	0.05 ± 0.02	8.2 ± 3.4	8.3 ± 3.6	23.8 ± 9.9	49.5 ± 21.3

a Errors include the uncertainty in the estimation of ^222^Rn activity of the fresh groundwater endmember.

## 4 Discussion

### 4.1 Nutrient Composition and Reactivity in Pristine vs Semiurban Subterranean Estuaries

Continental groundwaters of the semiurban sites of Baiona Bay contained several times more DIN than the local seawater (Table 1), converting fresh SGD into a potential but overlooked source of nitrogen to the Ría de Vigo.^29^ Attending to urban and agricultural land uses in the area surrounding the study sites, the low residence time characteristic of local aquifers, and the similar groundwater transit times between the terminus of the coastal aquifer and the studied beaches, the 2 orders of magnitude higher DIN concentrations and the different DOM composition (see Section 4.2) in the semiurban Baiona Bay wells compared to the pristine springs from Cíes Islands suggest a land use influence on groundwater composition. Baiona Bay DIN levels remain below those reported for aquifers in NW Portugal heavily impacted by agricultural pollution (900–1200 μM^42^). Yet, anthropogenic contamination cannot be excluded, as the short residence times within crystalline aquifers of the NW Iberian Peninsula limit DIN accumulation.^31^

Groundwater-borne DIN was only reflected in Panxón Beach porewaters, where a gravel layer originated from the weathering of nearby human-made structures promoted its internal oxygenation.^27^ The oxygenation prevented the anaerobic removal of groundwater-borne DIN and further enhanced DIN concentrations through DOM remineralization and nitrification. Yet, Calvo-Martin et al.^27^ showed that this groundwater-borne DIN was later removed at shallow sediments, where POM accumulates, via denitrification. The removal of groundwater-borne DIN in Panxón and the in situ production of DIN in Ladeira result in higher DIN exported toward the coast from Ladeira compared to Panxón in spring. DIN levels at Figueiras subterranean estuary were intermediate between those of Panxón and Ladeira, as it was their degree of oxygenation. Given the similar sediment organic matter content (Kruskal–Wallis *p* > 0.05), differences in dissolved oxygen are likely driven by porewater residence times. The sediment size distribution was used to calculate the beach permeabilities, following Krumbein and Monk.^43^ Apparent longer residence times in Ladeira subterranean estuary seem to be associated with reduced permeability (6.8 (0.5) × 10^–8^ cm^2^) compared to the permeability of Figueiras (5.2 (1.1) × 10^–7^ cm^2^). This is consistent with the lack of significant correlation between ^222^Rn and salinity at Ladeira (Spearman’s *p* > 0.05), which further suggests relatively longer residence times (allowing for significant ^222^Rn decay and production) in Ladeira compared to Figueiras (Kruskal–Wallis *p* < 0.05). Moreover, the Ladeira beach profile was flatter than that of Figueiras, suggesting longer flow paths along the upper saline plume.^44^ Higher residence times in Ladeira Beach seem to have favored dissolved oxygen consumption and DIN removal, whereas lower porewater residence times maintained O_2_ and promoted nitrification in Figueiras Beach, as suggested by the strong and negative correlation between NH_4_^+^ and both O_2_ and NO_3_^–^. DIN fluxes from the three subterranean estuaries were within the range observed in sandy beaches (median of 69.6 μmol m^–2^ hour^–1^;^17^).

As with DIN, DIP concentrations and apparent production across beaches appear linked to oxygenation, as the median values at the pristine site were between those of the semiurban sites. Assuming similar coastal organic carbon input and thus similar DIP production, differences in DIP levels across the subterranean estuaries seem to be mainly controlled by sorption onto metal oxides and carbonates.^45,46^ DIP-enrichment relative to the Redfield DIN/DIP ratio dominated both the anoxic semiurban and the pristine subterranean estuaries. This reflects intense DIN removal occurring together with coastal organic matter processing, likely driven by denitrification or Anammox, which is further supported in Ladeira through the deviation from conservative mixing. The elevated turnover of organic matter might result from a large input of marine labile organic matter associated with the characteristic high productivity of the coastal upwelling system, where these subterranean estuaries are located.^47^ This is reflected in the 2 orders of magnitude higher DIP fluxes than the global median estimations for sandy beaches (0.9 μmol m^–2^ hour^–1^;^17^). The differences in N and P processing between surveys in Ladeira might reflect an enhanced POM turnover in summer due to higher temperatures (Figure S1) or to higher residence times due to calmer wave conditions.^27^

Sediment organic C/total N ratios below Redfield suggest additional N removal through sorption onto Panxón and Figueiras sands.^48^ In other subterranean estuaries worldwide, N is often enriched in relation to P mainly due to anthropogenic N inputs to groundwaters and the low mobility of phosphorus. This typically alleviates nitrogen limitation of phytoplankton, enhancing coastal primary production.^18,49^ Instead, SGD from Ladeira and Figueiras subterranean estuaries might intensify nitrogen and silicate limitation of coastal primary producers.

The higher DSi levels in Baiona Bay wells compared to Cíes springs, translated into a higher DSi content in Panxón and Ladeira compared to Figueiras porewaters, were expected due to its larger catchment area.^50^ DSi levels and fluxes in Baiona Bay were still low compared to those at similar geological settings (77–687 μM and 414.2 μmol m^–2^ hour^–1^;^16,17^ ), which are likely explained by the fast recharge–discharge rates of the regional aquifers.^31^ The smaller watershed of the northern Island also explains the median higher salinities and the lower discharge rates of Figueiras Beach. Although land use impacts DSi levels in groundwaters,^51^ the differing watershed sizes prevent a direct assessment of anthropogenic impacts here. DSi was additionally produced along the flow path of the subterranean estuaries, most likely because of the dissolution of silicate-bearing minerals, although other studies have also detected potential Si precipitation in other subterranean estuaries.^52^

The DIN/DSi ratio showed greater variability within the same beach than the DSi/DIP ratio, indicating that DIN modulation might be more sensitive to internal redox gradients and DOM and POM supplies than DIP. This is in accordance with the opposite behavior of the different subterranean estuaries, with apparent production or consumption patterns of NH_4_^+^ and NO_3_^–^ depending on the site and the circulation zone. This contrasts with the more consistent apparent DIP production. Oxygenation thus plays a key role in the attenuation of anthropogenic nitrogen in subterranean estuaries, as previously suggested.^53^

The high dependence of nutrient ratios over sediment oxygenation, here linked to sediment permeability, highlights the importance of anthropogenic impacts over the geological matrix of subterranean estuaries (e.g., sand reworking, land reclamation). The expansion of the upper saline plumes, the main oxygenation mechanism within the subterranean estuaries, and the increase in sediment permeability might promote a shift toward increased N enrichment even at subterranean estuaries with high organic carbon turnover, subsequently enhancing coastal primary production.

### 4.2 DOM Composition and Reactivity in Pristine vs Semiurban Subterranean Estuaries

DOM at the semiurban wells of Baiona Bay exhibited a higher molecular weight (lower –S_275/295_) than at the pristine Cíes Islands, while their DOM content, aromaticity (SUVA_320_), and bioavailability (peak T/DOC) remained similar. The observed difference in DOM molecular weight might be related to the different land cover and maturity of the soils above the aquifer of the National park as observed elsewhere^54,55^ . DIN enrichment at Baiona Bay groundwaters might also stimulate organic carbon processing inland, as it occurs in other subterranean estuaries.^21,22^ Together with the larger residence times suggested by the elevated DSi levels observed,^50^ this might contribute to explain the apparent groundwater-borne DOM removal from the groundwaters entering the human-influenced beaches compared to the apparent production detected at the pristine site (Figure 1).

The maxima in DOC and peak T observed at depth in Figueiras Beach (Cíes Islands) might be explained by the remineralization of a buried organic layer, possibly from the 2002 Prestige oil spill.^56^ This also explains the high peak T there, as its fluorescence resembles that of naphthalene and benzene.^57^ However, the influence of this organic-rich horizon on porewater DOM composition seems restricted to the lower intertidal (St1; Figure S6 in Supporting Information). Calculations performed without St1 showed similar results as in Figure 1 (Table S6 in Supporting Information).

DOC consumption was detected in the upper saline plume of Figueiras Beach (Figure 1). This contrasts with Panxón, Ladeira, and other subterranean estuaries elsewhere, where coastal POM remineralization drives DOM accumulations in the upper saline plume (Figure 1;^11,13,14,53^ ). Flow-through experiments showed that shallow sediments of subterranean estuaries shift from producing to consuming DOM at high DOC concentrations (>400 μM) or when adding glucose.^21,58^ Porewater salinities at Figueiras upper saline plume resembled those at nearby seawaters, suggesting little contribution of the abundant terrestrial, groundwater-borne DOM there. Instead, coastal DOM consumption at Figueiras Beach likely results from the lability of the surrounding coastal seawater, in which peak T/DOC doubled the typical values of the Ría de Vigo seawater for each season (Table 1;^59^). Labile DOM can stimulate microbial activity to degrade recalcitrant terrestrial DOM through a priming effect.^60,61^ The removal of coastal DOM within the upper saline plume decreased the transport of labile DOM toward the beach interior, which might reduce the potential consumption of terrestrial DOM in Figueiras porewaters. The estimated exported DOM was within the range observed in other sandy beaches (1–2700 μmol m^–2^ hour^–1^; e.g.,^13,62^ ).

Despite having a larger contribution of terrestrial DOM, DOM in Figueiras was less recalcitrant than that of Ladeira. This suggests that residence times exert a stronger control over the bioavailability of DOM of these subterranean estuaries than the supply of terrestrial DOM. In Panxón beach, the shorter residence times within the gravel layer not only reduced the accumulation of humic-like FDOM but also enhanced the consumption of groundwater-borne DOM by injecting coastal, labile protein-like DOM toward the beach interior.^14,61^ Tidally driven oxygenation, together with the pumping of marine-derived labile organic matter, further enhanced by the human-derived gravel layer, may also stimulate the degradation of complex anthropogenic organic compounds, including contaminants of emerging concern (e.g.,^63^).

## 5 Conclusions

Nutrients and organic carbon discharged from the pristine Figueiras Beach and the semiurban oxygenated Panxón and anoxic Ladeira beaches were controlled by the intense biogeochemical reactivity of their respective subterranean estuaries. Beach porewater redox conditions and flushing times had a greater impact over the final solutes exported than the composition of their continental groundwaters. However, the human-derived alteration of the permeability of the sediment matrix (i.e., the gravel layer in Panxón beach) modified the water residence times and favored its internal oxygenation. Lower residence times reduce the quantity but increase the bioavailability of the DOM exported from the beach, benefiting the microbial heterotrophic community of the nearby surface seawaters. On the other hand, increased oxygenation promotes higher DIN/DIP ratios in the discharged groundwaters compared to those with lower oxygen. In DIN-limited coastal ecosystems such as the Ría de Vigo,^56^ the higher oxygenation of subterranean estuaries might be beneficial for coastal phytoplankton. However, this nutrient source may not be utilizable by diatoms due to the low DSi content relative to DIN and DIP discharged. Instead, the discharged groundwaters might benefit local dinoflagellates or cyanobacteria. Better constraints of porewater fluxes will be required to adequately assess the impacts of SGD in the Ría de Vigo at an ecosystem scale. This study highlights that the anthropogenic footprint on nutrient and organic carbon cycling in subterranean estuaries extends beyond human-derived solute enrichment of terrestrial groundwaters.

## Supplementary Material



## Data Availability

The data that support this study will be uploaded to DigitalCSIC online institutional repository upon acceptance (digital.csic.es).

## Notes

Additional details on the Cíes Islands survey (Text S1); analytical methods (Text S2); calculations of the conservative mixing lines (Text S3), transit times (Text S4), and groundwater discharge rates (Text S5); daily rainfall and temperatures during the surveys (Figure S1); detailed sampling design, measured salinities, and oxygen at Panxón and Ladeira subterranean estuaries (Figure S2); circulation zone classification (Figure S3); salinities and ^222^Rn activities at Figueiras Beach (Figure S4); mean deviation from mixing of a320, peak T, and peak C at Panxón, Figueiras, and Ladeira Beach (Figure S5); deviation from mixing of DOC and peak T in the vertical profiles of Figueiras Beach (Figure S6); median and range of transit times (Table S1); comparison of the deviation from conservative mixing when using different terrestrial endmembers (Table S2); median salinity, ^222^Rn activities, C-normalized peak T, SUVA_320_, and molecular weight (Table S3); median and range of salinities for each circulation zone, beach, and survey (Table S4); median and range of NH_4_^+^ and NO_3_^–^ (Table S5); comparison of mean deviation from mixing when including and excluding St1 at Figueiras Beach (Table S6) (PDF)
