# Supplementary material for: Human Influence on the Biogeochemical Reactivity of Subterranean Estuaries
Source: ACS ES T Water. 2026 May 18;6(6):3482–92. doi: 10.1021/acsestwater.5c01416 (PMC13270510; doi:10.1021/acsestwater.5c01416)
Supplement: Supplementary file 1 [file ew5c01416_si_001.pdf]

Supporting Information

Human influence on the biogeochemical reactivity of subterranean estuaries

Elisa Calvo-Martin[1,2], Xosé Antón Álvarez-Salgado[1], Valentí Rodellas[3], María José Pazó Fernández[1], Vanesa Vieitez Dos Santos[1] and J. Severino P. Ibánhez[1*]*

[1]Organic Geochemistry Lab, Department of Oceanography, Instituto de Investigacións Mariñas, Consejo Superior de Investigaciones Científicas (CSIC). Rua Eduardo Cabello 6, Vigo, 36208, Spain.

[2]PhD Program in Marine Science, Technology and Management, Universidade de Vigo. Edificio de Ciencias Experimentales, Vigo, 36310, Spain

[3]Department of Physics. Universitat Autònoma de Barcelona. Edifici C. Bellaterra (Cerdanyola del Vallès), 08193, Spain

*Corresponding author: J. Severino P. Ibánhez (jseverino@iim.csic.es) and Elisa Calvo-Martin (ecalvo@iim.csic.es)

Contents of this file:

Text S1 to S5

Figures S1 to S6

Tables S1 to S6

Text S1. Details on the Cies Islands survey (pristine site)

Salinity was determined in-situ using a pre-calibrated multiparametric probe (YSI Professional Plus; ±0.1). Dissolved oxygen samples were collected in 25 mL Winkler flasks and determined following the Winkler method (1). Samples for nutrients, DOC, CDOM and FDOM were filtered through 0.45 µm (Whatman, 25 mm of diameter) and kept frozen at -20ºC until analysis. Radon samples (10 mL) were collected using an air-tight syringe. After collection, radon samples were filtered (0.8 µm) and directly transferred to 20 mL vials prefilled with 10 mL of a high-efficiency mineral oil scintillation cocktail while minimizing water-air contact (2). During porewater extraction from the beach, the dead volume of the piezometer was discarded three times (< 100 mL) before starting sample collection. The sample volume collected from each piezometer was minimized to about 250 mL to reduce interferences between sampled depths.

Surface sediment samples were collected using a manual mini-corer (2.5 cm diameter, 10 cm length) at the upper and lower limits of the permanently saturated sediments in the location of the perpendicular transect. Each sediment core was sliced onsite into 0-2, 2-6 and 6-10 cm in the September survey and 0-1, 1-3, 3-6 and 6-10 cm in the May survey. These were collected in triplicate and equivalent depths from the same location where gently mixed to reduce spatial heterogeneity.

Text S2. Analytical methods

Salinity was measured in an AUTOSAL salinometer (±0.002) in Baiona Bay surveys. Dissolved oxygen was measured using the spectrophotometric Winkler method (<2 µM, standard deviation of 0.45%, 1). Nutrients (NO_3^-, NO_2^-, DSi and DIP) were measured on an Alliance Futura segmented flow autoanalyser following standard colourimetric methods (detection limits of 0.05, 0.02, 0.05 and 0.02 µM, respectively; 3) and and NH_4^+ following the fluorimetric method (detection limit of 0.04 µM; 4). CDOM spectrum from 700 to 250 nm of each sample was measured in a Beckham Coulter DU 800 spectrophotometer (detection limit of 0.02 m^{-1}). FDOM Peak C (terrestrial humic-like substances) and Peak T (protein-like material) were recorded at excitation/emission wavelengths of 340/440 and 280/350 nm, respectively, in a Perkin Elmer LS55 spectrofluorometer (detection limits of 0.0003 r.u. and 0.0004 r.u., respectively; 5–7). Samples for CDOM and FDOM were measured in a 1 cm quartz cuvette at room temperature (25ºC). Baseline corrections were applied to CDOM by subtracting the CDOM spectrum from the average absorbance between 600 and 700 nm. CDOM was transformed to Napierian absorption coefficient by multiplying by 2.303 and dividing by the cuvette optical path. FDOM intensities were normalized to the Raman peak area and absorbance was used to correct for inner filter effects (8,9). After measuring CDOM and FDOM, the remaining volume was acidified to pH<2 and de-bubbled with high-purity N_2 to remove CO_2. Then, DOC and total dissolved nitrogen (TDN) were measured following the high-temperature catalytic oxidation method in a Shimadzu TOC-V analyser connected in line to a nitrogen chemiluminescence measuring unit TNM-1 (detection limit of DOC: 1-2 µM; 10). ^{222}Rn activities were measured by liquid scintillation counting on a Quantulus 1220 with alpha-beta discrimination counting during Cíes Islands surveys

(background of 0.22–0.4 cpm; efficiency of 3.0±0.9). For Baiona Bay surveys, a Rad7 Radon detector with a RadH2O accessory was used (Durridge Company, Inc.; detection limit, precision, and accuracy of 36 Bq m^{-3}, 12 Bq m^{-3}, and 91%, respectively; 11). The comparability of these two methods has been recently demonstrated (12). During Baiona Bay surveys, ^{222}Rn was measured within 2 days after collection. Internal decay corrections were applied.

Sediment grain size was measured in a laser particle size analyser (Beckham Coulter; sediment fraction <2 mm). Porosity was calculated by dividing the volume of porewater by the volume of wet sediment. The volume of porewater was obtained after drying a sediment subsample at 60ºC until constant weight and by correcting with the salts left in the dried sample. The percentage of sediment organic matter was determined by loss-on-ignition (13). To obtain the carbonate content and sediment organic C/total N ratio, sediment samples were powdered using a laboratory ball mill with a natural agate tank for 10-15 min at medium intensity. Subsamples for sediment organic C and total N content were exposed to HCl flumes for 6 h to remove inorganic C. Then, a CHN elemental analyzer Perkin Elmer 2400 A was used to measure both non-acidified and acidified samples. Carbonate sediment content was obtained by subtracting the resulting sediment total C from sediment organic C.

Text S3. Calculation of the conservative mixing concentrations

We evaluated the chemical reactivity within the subterranean estuaries by comparing the measured NO_3^-, NH_4^+, HPO_4^{2-}, DOC, a_{320}, peak C and peak T at the porewaters of each survey with the conservative mixing concentrations using different terrestrial endmembers for each circulation zone and as marine endmember the nearby seawaters. The absorption coefficient a_{320} as a proxy of CDOM concentration in replacement of the commonly used absorption coefficient at 254 nm (a_{254}) due to possible interferences between a_{254} and the H_2S detected during Ladeira surveys. Porewater salinities and radon activities were used as conservative tracers. For the freshwater discharge tube, we used as terrestrial endmembers the F3 spring for Figueiras beach and F4 well for Panxón and Ladeira beaches. These wells were chosen over the others because of their proximity to the sampled beach. Similar results were obtained when using F2 for Figueiras beach and F5 for Panxón and Ladeira beaches (Table S3). F1 was discarded as a potential terrestrial endmember as its ^{222}Rn activities were below those observed in Figueiras beach. For the upper saline plume, we used as terrestrial endmember the sample with highest radon activity or the lowest salinity found at each circulation zone and survey, and as marine endmember the seawater sample collected at each survey. Conservative mixing concentrations (C_{mix}) for the two types of mixing curves are calculated using a linear mixing model, where the slope (m) and the intercept (n) are given by

$$m = (C_{terr} - C_{mar})/(T_{terr} - T_{mar}) \quad (1)$$

$$n = C_{terr} - m\,T_{terr} \quad (2)$$

and the conservative mixing concentration is

$$C_{mix} = m\,T_{pw} + n \qquad (3)$$

Where C refers to the target solute concentration, T to the tracer concentration (salinity or radon), the suffix "terr" to the selected terrestrial endmember, the suffix "mar" to the marine endmember and "pw" the measured porewater concentrations.

Once the conservative mixing concentrations were calculated, they were subtracted to the measured concentrations to obtain residual concentrations, that will be used to gain insight on the biogeochemical reactivity within the beach. ^{222}Rn was not used as a circulation tracer in Ladeira beach due to possible interferences with H_2S detected during sampling (14).

Text S4. Calculation of transit times

Sediment samples collected during the 2019 and 2023 surveys at Panxón and Ladeira beaches were used to estimate porewater ^{222}Rn equilibrium activities (i.e. maximum activity sustained by sediment ^{226}Ra decay; Rn_{eq}). These were determined through sediment incubations (200 g to 1 kg dry weight; n = 6 per beach) in Ra-free water (Colbert and Hammond, 2008). Equilibrium ^{222}Rn activities in the incubation water were determined after >40 days of incubation using a Rad7 Radon detector, following the same procedure applied for porewater ^{222}Rn measurements during the Baiona Bay surveys. Mean ± standard deviation ^{222}Rn activities were 5613 ± 1421 Bq m^{-3} in Ladeira and 5426 ± 1236 Bq m^{-3} in Panxón. These are similar to those found by Ibánhez et al. (11) using sediment samples distributed throughout the entire embayment. Thus, mean Rn_{eq} of Panxón and Ladeira was used for Figueiras Beach (5506 ± 1203 Bq m^{-3}).

Transit times were calculated using ^{222}Rn activities from nearby sampled wells and the excess ^{222}Rn activities measured in each beach (Rn_{excess}). Excess ^{222}Rn activities were calculated by subtracting both the measured ^{226}Ra activities and the sediment-water equilibrium ^{222}Rn activities (Rn_{eq}) from the measured porewater ^{222}Rn activities. In Baiona Bay samples, ^{226}Ra activities in porewater samples were determined from samples stored for >30 days, allowing ^{222}Rn and ^{226}Ra to reach secular equilibrium. ^{226}Ra activities were determined with a Rad7 as ^{222}Rn. In the northern Cies Island, porewater samples for ^{226}Ra were collected and analysed following Rodríguez-Puig et al. (15). Briefly, samples were filtered through a column loaded with 20 g of MnO_2^--impregnated acrylic fiber (16). Mn-fibers were cleaned with MilliQ ultra-pure water and incinerated at 820 °C. The resulting ashes were placed into sealed vials and aged for a minimum of 21 days to achieve radioactive equilibrium. ^{226}Ra was analyzed via gamma spectrometry, using a well-type high-purity Ge detector.

Transit times (t) were then calculated as follows:

$$t = -\ln\left(\frac{Rn_{excess}/\%_{freshgw}}{Rn_{aquifer}}\right)/\lambda$$

Where $\%_{freshgw}$ is the proportion of fresh groundwater, calculated as 1 minus the ratio between the measured porewater salinity and the maximum porewater salinity observed at each beach and survey. $Rn_{aquifer}$ represents the ^{222}Rn activities measured in nearby wells, and λ is the decay constant of ^{222}Rn (0.18 day^{-1}). Only porewater samples with salinity values below the median salinity of each survey and beach were used to estimate transit times.

Text S5. Calculation of groundwater discharge rates

The ^{222}Rn measured in the porewater samples at 10 and 28 cm depth were used to estimate the porewater residence times through ^{222}Rn disequilibrium as follows ([17]):

$$\tau = -\frac{1}{\lambda}\ \ln\left(1 - \frac{Rn_s}{Rn_{eq}}\right)$$

Where λ is the decay constant of ^{222}Rn (0.181 d^{-1}) and ^{222}Rn equilibrium activity (Rn_{eq}) is the maximum activity sustained by sediment ^{226}Ra decay. The ^{222}Rn activities measured inside the beaches include both the ^{222}Rn activities produced by the decay of the ^{226}Ra present in the sands (Rns), the ^{222}Rn produced by the decay of ^{226}Ra transported by groundwater and the ^{222}Rn transported from the fresh groundwater. As porewater samples in the studied subterranean estuaries are affected by radon-enriched fresh SGD, Rn_s was calculated as:

$$Rn_s = Rn_{pw} - (Rn_{gw}\,\frac{\%_{gw}}{\%_{sw}}) - Ra_{pw}$$

where Rn_{gw} are the radon activities associated with the continental fresh groundwater endmember, Ra_{pw} the measured porewater ^{226}Ra activities and Rn_{pw} the measured porewater ^{222}Rn activities at Panxón, Ladeira and Figueiras subterranean estuaries. The percentage of groundwater ($\%_{gw}$) and seawater in the sample ($\%_{sw}$) were calculated with porewater salinities. Rn_{gw} was calculated by extrapolating to 0 salinity the ^{222}Rn activities from the samples which had a salinity below the 20% of the samples of each survey.

Due to the relatively low correlation between salinity and ^{222}Rn (Panxón Spearman's correlation: -0.65, $p<0.05$; Ladeira: $p>0.05$; Figueiras: -0.68, $p<0.05$), the ^{222}Rn activities of the freshwater endmember (Rn_{gw}) varied depending the sample used to calculate Rn_{gw}. To overcome the high uncertainty of Rn_{gw}, multiple fresh groundwater endmembers were tested. The Rn_s used here to calculate porewater residence times (τ) is the mean Rn_s obtained from each of the endmembers tested (i.e. from each Rn_{gw} obtained from the samples which salinities were below the 20% of the samples of each survey), and the error associated to Rn_s is the standard error of the mean. Seawater endmember was considered to have 0 ^{222}Rn activities.

Assuming vertical porewater flow, discharge rates were estimated by dividing the sampling depth by the mean residence time of porewater in shallow sediments (<30 cm) and multiplying by sediment porosity. The number of samples used for these estimations varied among beaches and surveys (n), reflecting the uncertainty associated with Rn_s. Fluxes of DIN, DIP, DSi, and DOC were calculated by multiplying discharge rates by the corresponding solute concentrations measured in

the samples where the discharge rates could be determined. The standard errors of the mean were smaller than the uncertainties associated with Rn_s; therefore, only the latter are reported in Table 2.

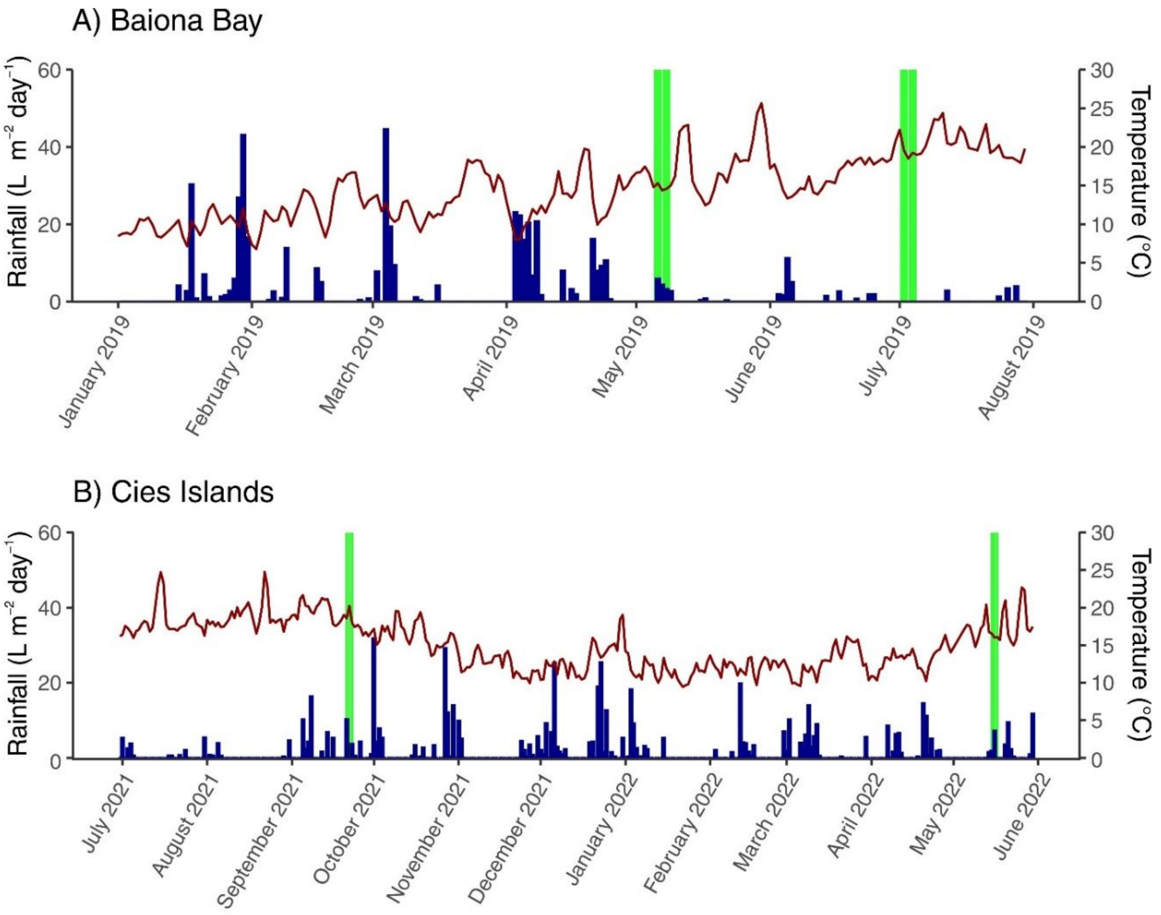

Figure S1. Daily rainfall (blue bars) and air temperatures (red lines) at 1.5 m during (A) the 2018-2019 period surveyed in Baiona Bay and (B) during the 2021-2022 period surveyed in the northern Cies Island. Surveyed periods are indicated in green. The temperatures during the spring and summer surveys were 15.9°C and 19.1°C in Baiona Bay, and 16.2°C and 18.9°C in the Cies Islands, respectively. The accumulated rainfall from 40 days before each survey was 168.6 and 27.1 L m^{-2} day^{-1} in spring and summer during Baiona Bay surveys, and of 70.6 and 65.9 L m^{-2} day^{-1} during the Cies Island survey, respectively. Data was obtained from the Port of Vigo and Cíes meteorological stations from Meteogalicia repository (meteogalicia.gal).

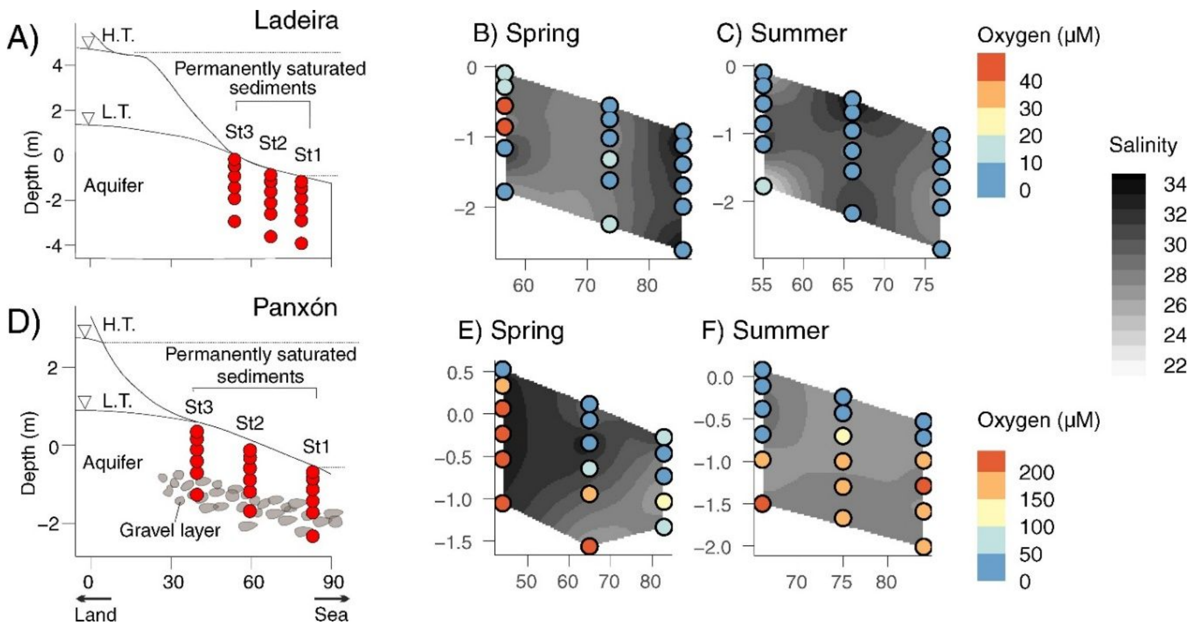

Figure S2. Position of the stations and depths of the samples collected with piezometers in Ladeira (A) and Panxón (D). Salinity (white to black) and dissolved oxygen concentrations (blue to red) measured in each survey and beach are shown in B and C for Ladeira, and in E and F for Panxón. Note that the oxygen color scale is different for each beach. The gravel layer in Panxón beach promoted internal oxygenation and the transport of DIN as NO_3^-, contrasting with the anoxic conditions within Ladeira beach.

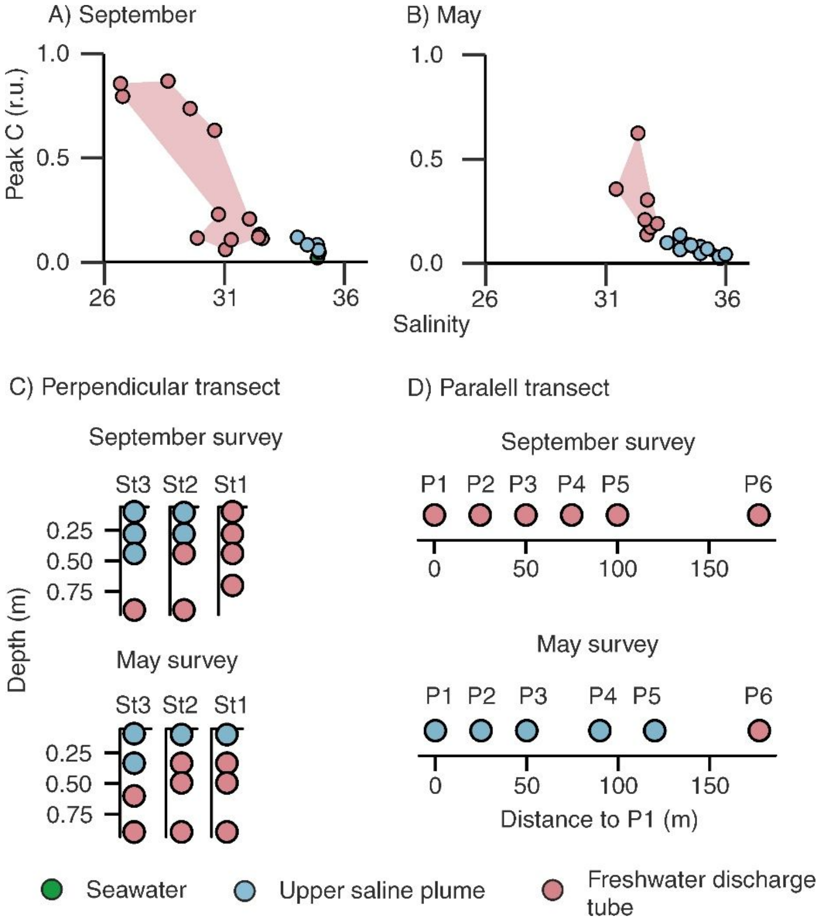

Figure S3. A and B) Peak C vs salinity of each survey performed in Figueiras beach, together with the nearby seawater sample. Peak C was used here as continental groundwater tracer due to the strong and opposite correlation between peak C and salinity (September ρ: -0.73; May: -0.95), and peak C and ^{222}Rn (September ρ:0.88; May ρ: 0.73). C and D) Classification of the porewater samples from Figueiras Beach into the circulation zones from Robinson et al. (18) at each transect and survey. Samples were classified into the upper saline plume when their salinities were above 33.5, and in the freshwater discharge tube when they were below. The salt-wedge was not observed during the surveys of Figueiras beach (Figure S3).

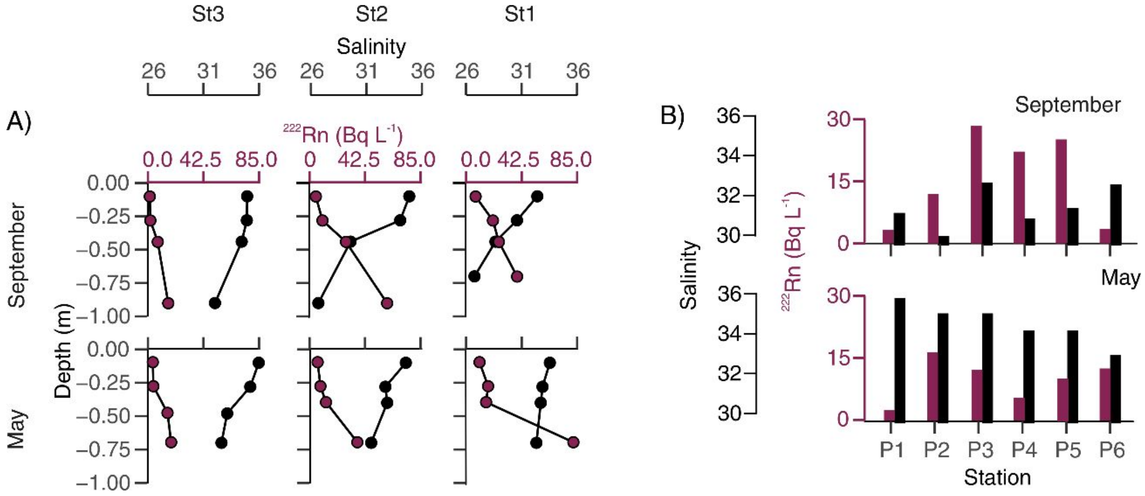

Figure S4. A) Distribution of salinity (black) and ^{222}Rn activities (purple) in depth along the three piezometer stations (St1, St2 and St3) from the transect perpendicular to the coastline performed in Figueiras Beach in September (upper panels) and May (lower panels) surveys. B) Distribution of salinity (black) and ^{222}Rn activities (purple) along the six piezometer stations (P1-P6) from the transect parallel to the coastline performed in Figueiras Beach in September (upper panels) and May (lower panels) surveys. Higher salinities in spring porewaters compared to summer suggest a higher contribution of continental groundwater to summer beach porewaters. This is supported by a lower accumulated water balance the month before the spring survey (-70.2 L m^{-2}) compared to the summer survey (-57.4 L m^{-2}; meteogalicia.com). ^{222}Rn and salinities did not show any apparent correlation in the parallel transect, suggesting diverse terrestrial endmembers within the beach. In this study, we evaluated the chemical reactivity of Figueiras subterranean estuary by assuming that the chemical composition of these terrestrial endmembers was homogeneous.

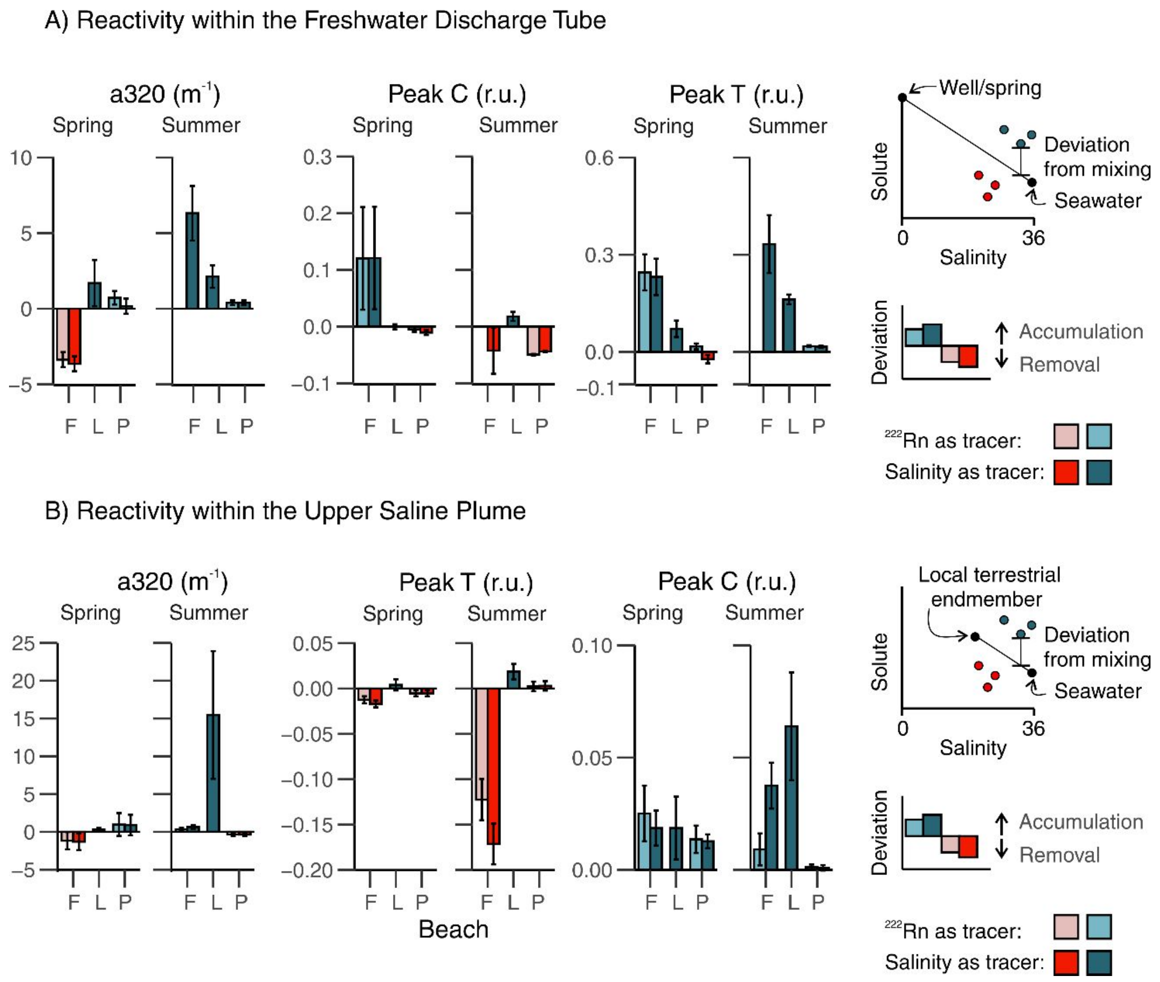

Figure S5. Mean deviation from mixing between seawater and the terrestrial endmember of each circulation zone at Figueiras, Ladeira and Panxón Beaches calculated with radon (light colours) and salinity (dark colours) for (A) the freshwater discharge tube and (B) the upper saline plume of a320, peak T and peak C. Error bar represents the standard error.

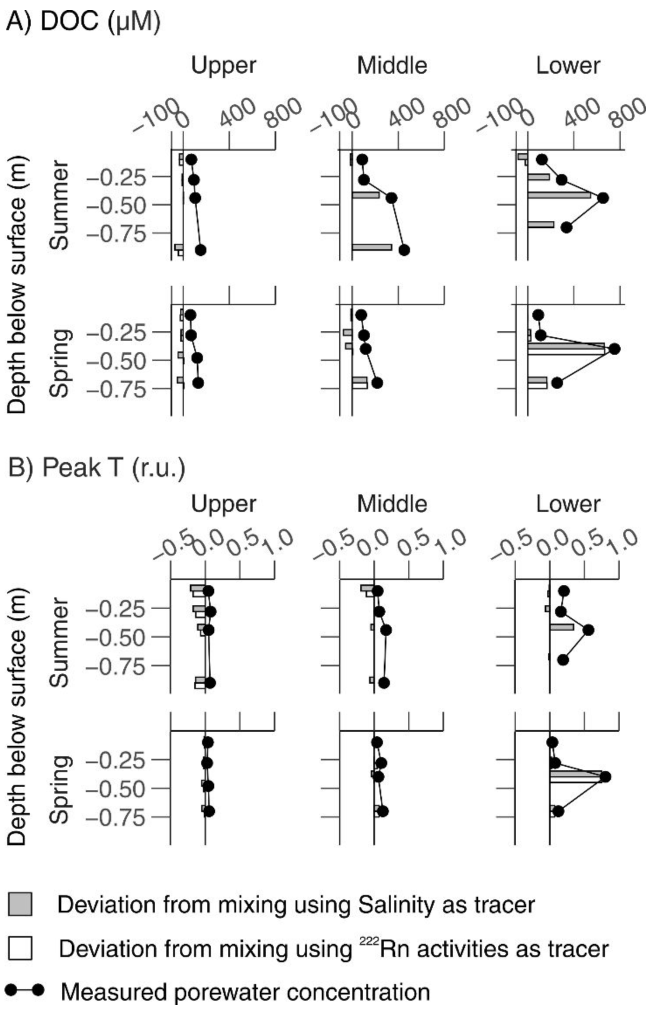

Figure S6. Deviation of measured porewater DOC (A) and peak T (B) from the conservative mixing between the terrestrial local endmember from each circulation zone and seawater samples, using radon activities (white) and salinities (grey), at the vertical profiles of the perpendicular transect of Figueiras Beach. Measured solute concentrations in every vertical profile from the perpendicular transect are represented by dots and lines.

Table S1. Median and range of transit times in the three study sites, together with the number of porewater samples used to obtain these estimations per beach, season and well. The ^{222}Rn activities from F1, F2 and F3 were used for Figueiras beach, and F4 and F5 for Ladeira and Panxón beaches.

	Spring			Summer		
Well	**F1/F4**	**F2/F5**	**F3**	**F1/F4**	**F2/F5**	**F3**
Figueiras		21.3 (18.9 to 26.1; n=5)		4.8 (1.1 to 8.4; n=3)	20.7 (11.3 to 30.6; n=8)	29.2 (19.8 to 39.0; n=8)
Ladeira	15.4 (9.5 to 23.7; n=7)	22.9 (17.0 to 31.3; n=7)		17.6 (14.4 to 26.9; n=5)	22.6 (19.3 to 31.8; n=5)	
Panxón	22.3 (n=1)	29.9 (n=1)		25.7 (23.5 to 32.4; n=4)	30.6 (28.5 to 37.4; n=4)	

Table S2. Comparison of the deviation from conservative mixing when using as terrestrial endmembers the springs F2 and F3 for Figueiras beach and the wells F4 and F5 for Panxón and Ladeira beaches for evaluating the freshwater discharge tube reactivity. F1 was not used as a possible terrestrial endmember of the freshwater discharge tube samples of Figueiras beach because of its low ^{222}Rn activities, below those observed in Figueiras porewaters. Results are expressed as the mean deviation from mixing of the zone, season and beach together with the standard error.

Beach	Well	Season	DOC using ^{222}Rn (µM)	DOC using ^{222}Rn (µM)	NO_3^- using ^{222}Rn (µM)	NO_3^- using Salinity (µM)	DIP using ^{222}Rn (µM)	DIP using Salinity (µM)
Panxon	F4	Summer	-47.4 ± 1.2	-41.8 ± 1.3	1.8 ± 12.3	-28.6 ± 12.4	1.6 ± 0.7	0.8 ± 0.6
		Spring	-30.0 ± 5.2	-40.8 ± 6.7	37.8 ± 16.5	25.7 ± 15.4	2.0 ± 0.1	1.7 ± 0.1
	F5	Summer	-57.8 ± 1.3	-67.9 ± 1.1	35.5 ± 12.9	-44.3 ± 12.5	2.9 ± 0.7	3.1 ± 0.6
		Spring	-25.6 ± 5.1	-30.7 ± 5.6	37.5 ± 16.5	4.7 ± 14.5	1.9 ± 0.1	0.8 ± 0.2
Ladeira	F4	Summer	-4.3 ± 4.7	-0.8 ± 4.9	-59.8 ± 7.0	-76 ± 8.4	14.8 ± 4.2	14.3 ± 4.4
		Spring	-23.4 ± 2.5	-30.7 ± 3.4	-1.3 ± 10.4	-9.6 ± 9.5	8.6 ± 2.4	8.4 ± 2.4

	F5	Summer	-18.1 ± 5.7	-26.0 ± 6.2	-18.1 ± 1.8	-91.1 ± 10.1	16.4 ± 4.3	16.5 ± 4.3
		Spring	-17.0 ± 2.9	-21.0 ± 3.1	-1.7 ± 10.5	-29.9 ± 9.5	8.4 ± 2.4	7.5 ± 2.4
Figueiras	F2	Summer	128.0 ± 47.8	118.1 ± 47	28.6 ± 11.1	27.1 ± 11.2	3.4 ± 0.4	3.1 ± 0.4
		Spring	136.9 ± 77.6	135.9 ± 77.7	22.1 ± 11.6	22.0 ± 11.5	4.0 ± 0.5	4.0 ± 0.5
	F3	Summer		126.4 ± 47.7		29.0 ± 11.1		3.4 ± 0.4
		Spring	140.1 ± 77.7	137.3 ± 77.7	22.8 ± 11.5	22.9 ± 11.5	4.1 ± 0.5	4.1 ± 0.5

Table S3. Median salinity, ^{222}Rn activities, C-normalized peak T (peak T/DOC), SUVA$_{320}$ and molecular weight (-S$_{275/295}$) with its range of values.

	Salinity	^{222}Rn Activities (Bq/L)	Peak T/DOC ($x10^{-4}$ r.u./µM)	SUVA$_{320}$ (L/mg C m)	-S$_{275/295}$ ($x10^{-3}$ nm^{-1})
**Seawater**					
Northern Cíes Islands (n=2)	35.2 (34.7 to 35.8)	0.1 (0 to 0.2)	128.6 (63.3 to 193.8)	26.2 (24.8 to 27.6)	19.0 (14.1 to 24.0)
Baiona Bay (n=4)	34.5 (33.3 to 34.6)	0.1 (0 to 0.3)	36.8 (27.5 to 86.7)	27.5 (23.2 to 29.1)	22.8 (21.9 to 24.2)
**Continental groundwaters**					
Northern Cíes Islands (n=6)	0.4 (0.3 to 0.7)	359.7 (5.4 to 1645.5)	33.7 (28.7 to 61.6)	179.2 (6.9 to 234.5)	15.9 (14.4 to 18.2)
Baiona Bay (n=4)	0.0 (0 to 0.1)	63.5 (6.0 to 202.6)	43.0 (37.6 to 44.7)	170.9 (136.1 to 202.9)	11.9 (9.9 to 14.3)
**Upper Saline Plume**					
Figueiras (n=14)	34.9 (33.5 to 36)	5.9 (1.4 to 16.5)	46.6 (24.3 to 82.9)	83.9 (46.6 to 143.6)	15.9 (9.3 to 20.7)
Ladeira (n=13)	29.6 (27.9 to 32.7)	4.1 (2.5 to 8.1)	47.5 (23.5 to 72.7)	126.8 (81.7 to 230.8)	6.9 (0.1 to 16.4)
Panxon (n=14)	31.1 (27 to 32.5)	4.3 (1.5 to 7.5)	37.8 (24 to 64.9)	89.9 (60.5 to 139.8)	7.7 (2.1 to 32.4)
**Freshwater discharge tube**					
Figueiras (n=21)	32.0 (26.7 to 33.1)	17.8 (3.3 to 82.1)	57.0 (31.5 to 423.7)	137.1 (40.3 to 245.7)	14.0 (7.1 to 30.3)

Ladeira	27.7	7.3	54.6	224.5	16.3
(n=15)	(22.9 to 28.7)	(3.3 to 12.3)	(23 to 84.7)	(98.2 to 449.5)	(3 to 27.1)
Panxon	26.7	5.7	49.0	114.8	8.9
(n=18)	(26 to 31.9)	(4.1 to 8.4)	(25.4 to 70.9)	(76.7 to 149.3)	(2.8 to 39.2)

Table S4. Median salinities (range) measured at each circulation zone and beach divided by survey.

	Freshwater discharge tube		Upper saline plume	
	Spring	Summer	Spring	Summer
Figueiras	32.7 (31.4 to 33.1)	30.8 (26.7 to 32.6)	34.9 (33.5 to 36)	34.9 (34 to 34.9)
Ladeira	27.6 (26.2 to 28.4)	28 (22.9 to 28.7)	29.1 (27.9 to 31.1)	29.9 (28.8 to 32.7)
Panxón	28 (26 to 31.9)	26.4 (26.2 to 27.4)	32.1 (28 to 32.5)	28 (27 to 28.6)

Table S5. Median NH_4^+ and NO_3^- with their range of values in the samples presented here. These were grouped into sweater, continental groundwater, upper saline plume and freshwater discharge tube samples.

	NH_4^+ (µM)	NO_3^- (µM)
**Seawater**		
Northern Cíes Islands (n=2)	1.2 (0.7 to 1.7)	1.7 (0.5 to 2.9)
Baiona Bay (n=4)	1.4 (0.3 to 2.4)	2.9 (1.2 to 3.6)
**Continental groundwaters**		
Northern Cíes Islands (n=2)	0.7 (0 to 1)	13.1 (0.2 to 18.7)
Baiona Bay (n=4)	1.9 (0.4 to 4.1)	273 (101.1 to 396.8)
**Upper saline plume**		
Figueiras (n=14)	0.4 (0 to 8.5)	31.7 (0.1 to 83.2)
Ladeira (n=13)	16.2 (0.1 to 40.3)	0.4 (0 to 55.4)
Panxon (n=14)	0.1 (0 to 18.9)	34 (0.5 to 99.7)
**Freshwater discharge tube**		
Figueiras (n=21)	4 (0 to 27.1)	3.8 (0 to 107.2)
Ladeira (n=15)	12.7 (0.1 to 18.4)	0.3 (0 to 77.5)
Panxon (n=18)	0.3 (0 to 14.7)	52.2 (0 to 110.6)

Table S6. Comparison of the mean deviation from conservative mixing of the freshwater discharge tube samples of Figueiras Beach when incorporating or excluding St1 from the calculations.

St1?	Season	DOC using ^{222}Rn (µM)	DOC using Salinity (µM)	Peak C using ^{222}Rn (r.u.)	Peak C using Salinity (r.u.)	Peak T using ^{222}Rn (r.u.)	Peak T using Salinity (r.u.)
No	Summer		74.1±42.5		0.236 ±0.096		-0.088 ±0.036
No	Spring	52.1±20.4	49.1±20.2	0.187 ±0.037	0.172 ±0.036	0.024 ±0.015	0.025 ±0.015
Yes	Summer		126.4±47.7		0.333 ±0.089		-0.042 ±0.041
Yes	Spring	140.1±77.7	137.3±77.7	0.245 ±0.056	0.232 ±0.056	0.12 ±0.091	0.121 ±0.091

References in Supplementary Materials

(1) Labasque, T.; Chaumery, C.; Aminot, A.; Kergoat, G. Spectrophotometric Winkler Determination of Dissolved Oxygen: Re-Examination of Critical Factors and Reliability. *Mar. Chem.* **2004**, *88* (1–2), 53–60. https://doi.org/10.1016/j.marchem.2004.03.004.

(2) Cable, J. E.; Martin, J. B. In Situ Evaluation of Nearshore Marine and Fresh Pore Water Transport into Flamengo Bay, Brazil. *Estuar. Coast. Shelf Sci.* **2008**, *76* (3), 473–483. https://doi.org/10.1016/j.ecss.2007.07.045.

(3) Grasshoff, K.; Kremling, K.; Ehrhardt, M. *Methods of Seawater Analysis. (Eds.)*; John Wiley & Sons., 1999.

(4) Kérouel, R.; Aminot, A. Fluorometric Determination of Ammonia in Sea and Estuarine Waters by Direct Segmented Flow Analysis. *Mar. Chem.* **1997**, *57* (3–4), 265–275. https://doi.org/10.1016/S0304-4203(97)00040-6.

(5) Coble, P. G. Characterization of Marine and Terrestrial DOM in Seawater Using Excitation-Emission Matrix Spectroscopy. *Mar. Chem.* **1996**, *51* (4), 325–346. https://doi.org/10.1016/0304-4203(95)00062-3.

(6) Nieto-Cid, M.; Álvarez-Salgado, X. A.; Gago, J.; Pérez, F. F. DOM Fluorescence, a Tracer for Biogeochemical Processes in a Coastal Upwelling System (NW Iberian Peninsula). *Mar. Ecol. Prog. Ser.* **2005**, *297*, 33–50. https://doi.org/10.3354/meps297033.

(7) Stedmon, C. A.; Nelson, N. B. The Optical Properties of DOM in the Ocean. In *Biogeochemistry of Marine Dissolved Organic Matter: Second Edition*; Academic Press, 2015; pp 481–508.

(8) Ohno, T. Fluorescence Inner-Filtering Correction for Determining the Humification Index of Dissolved Organic Matter. *Environ. Sci. Technol.* **2002**, *36* (4), 742–746. https://doi.org/10.1021/es0155276.

(9) Murphy, K. R.; Butler, K. D.; Spencer, R. G. M.; Stedmon, C. A.; Boehme, J. R.; Aiken, G. R. Measurement of Dissolved Organic Matter Fluorescence in Aquatic Environments: An Interlaboratory Comparison. *Environ. Sci. Technol.* **2010**, *44* (24), 9405–9412. https://doi.org/10.1021/es102362t.

(10) Álvarez-Salgado, X. A.; Miller, A. E. J. Simultaneous Determination of Dissolved Organic Carbon and Total Dissolved Nitrogen in Seawater by High Temperature Catalytic Oxidation: Conditions for Precise Shipboard Measurements. *Mar. Chem.* **1998**, *62* (3–4), 325–333. https://doi.org/10.1016/S0304-4203(98)00037-1.

(11) Ibánhez, J. S. P.; Álvarez-Salgado, X. A.; Nieto-Cid, M.; Rocha, C. Fresh and Saline Submarine Groundwater Discharge in a Large Coastal Inlet Affected by Seasonal Upwelling (Ría de Vigo, NW Iberian Peninsula). *Limnol. Oceanogr.* **2021**, *66*, 2141–2158. https://doi.org/10.1002/lno.11733.

(12) Natarajan, C.; Goswami, S.; Kuttiveettil Savy, B.; Parthasarathy, K.; Challa, V. S. Radon Measurement in Ground Water Samples Using RAD7 Monitor-Based Bubbler and Liquid Scintillation Counting – a Comparative Study. *J. Radioanal. Nucl. Chem.* **2026**. https://doi.org/10.1007/s10967-026-10740-3.

(13) Ball, D. F. Loss-on-Ignition as an Estimate of Organic Matter and Organic Carbon in Non-Calcareous Soils. *J. Soil Sci.* **1964**, *15* (1), 84–92. https://doi.org/10.1111/j.1365-2389.1964.tb00247.x.

(14) Begy, R.-C.; Savin, C.-F.; Timar-Gabor, A. Correction of the Effects of Carbon Dioxide and Hydrogen Sulfide on Electrostatic Cell Monitors Measurements of Radon in Water. *J. Environ. Chem. Eng.* **2022**, *10* (1), 107040. https://doi.org/10.1016/j.jece.2021.107040.
(15) Rodriguez-Puig, J.; Rodellas, V.; Diego-Feliu, M.; Alcolea, A.; Jiménez-Martínez, J.; Alorda-Montiel, I.; Alorda-Kleinglass, A.; Pereira, F.; Manzano, M.; Gilabert, J.; Garcia-Orellana, J. Seasonality of Submarine Groundwater Discharge Pathways in a Coastal Lagoon Revealed by Radium Isotopes: The Importance of Porewater Exchange in Summer. *J. Hydrol.* **2025**, *661*, 133616. https://doi.org/10.1016/j.jhydrol.2025.133616.
(16) Moore, W. S.; Reid, D. F. Extraction of Radium from Natural Waters Using Manganese-Impregnated Acrylic Fibers. *J. Geophys. Res. 1896-1977* **1973**, *78* (36), 8880–8886. https://doi.org/10.1029/JC078i036p08880.
(17) Goodridge, B. M.; Melack, J. M. Temporal Evolution and Variability of Dissolved Inorganic Nitrogen in Beach Pore Water Revealed Using Radon Residence Times. *Environ. Sci. Technol.* **2014**, *48* (24), 14211–14218. https://doi.org/10.1021/es504017j.
(18) Robinson, C.; Li, L.; Barry, D. A. Effect of Tidal Forcing on a Subterranean Estuary. *Adv. Water Resour.* **2007**, *30* (4), 851–865. https://doi.org/10.1016/j.advwatres.2006.07.006.
